# Adult male with non-resolving opacity in the right hemithorax

**DOI:** 10.11604/pamj.2014.19.339.5290

**Published:** 2014-12-01

**Authors:** Liaqat Ali Chaudhry, Marwan Zamzami, Asirvatham Alwin Robert

**Affiliations:** 1 Department of Internal Medicine, Pulmonary Division, Sultan Bin Abdul Aziz Humanitarian City, Riyadh, Saudi Arabia; 2 Department of Orthopedic Surgery, College of Medicine, King Saud University Riyadh, Saudi Arabia; 3 Department of Endocrinology and Diabetes, Diabetes Treatment Center, Prince Sultan Military Medical City, Riyadh, Saudi Arabia

**Keywords:** Right hemithorax, Opacity, Agenesis

## Abstract

Pulmonary agenesis is a well-known but rare congenital anomaly of the respiratory system. It represents failure of development of the primitive lung bud. We are reporting bilobar agenesis of the right lung associated with multisystem involvement in an adult patient.

## Introduction

Lung atresia or agenesis being an uncommon congenital condition represents development defect of the primitive lung bud. This was first time accidently detected by De Pozze [1] during medicolegal autopsy reported in 1673 on an adult female. Clinical based diagnosis was first made by Munch Meyer in 1885 [2]. Until 1977 more than 200 [3] cases has been reported and many more until recently [4]. From Saudi Arabia first case has been reported by Mohammed Al-anezi [5] in an adult female in 2006. Often one or few cases has been reported by authors, but major reviews are those of Oyamada et al [6], Vale [7], Maltz and Nadas [8] and Sbokaos and Mcmilllan [9]. This condition may be seen alone limited to the lung or may be associated with multisystem involvement. Age of presentation varies dependent on extent of lung involvement and variability of symptoms making diagnosis very challenging. There may be no symptoms and condition may be discovered incidentally associated with other co-morbidities. We present an adult having non resolving opacity in the right hemithorax.

## Patient and observation

**Figure 1:**
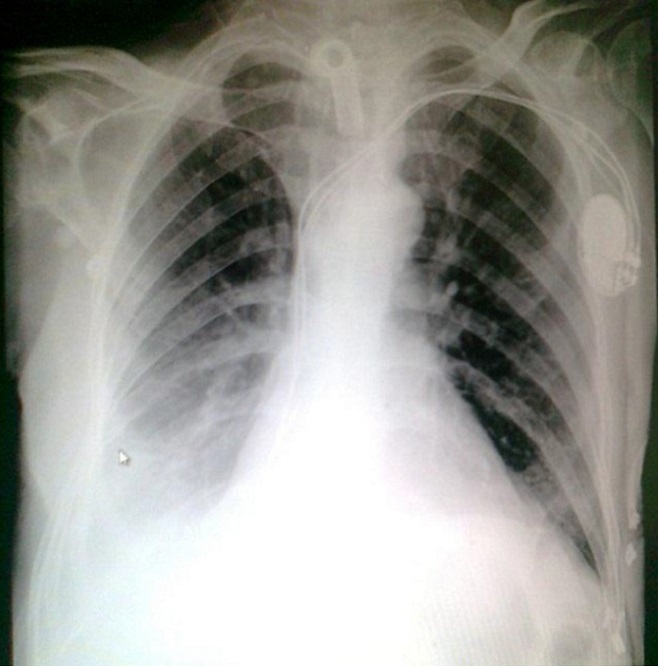
Chest x-ray

**Figure 2:**
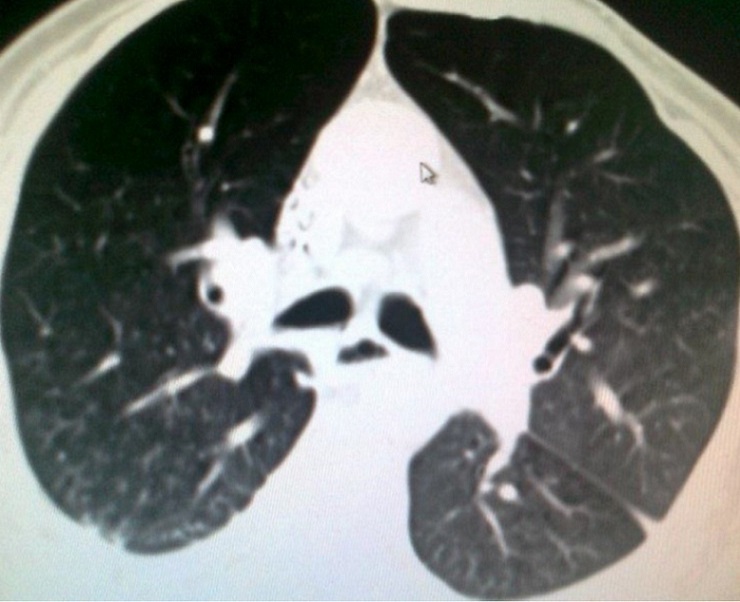
CT-scan chest with contrast

**Figure 3:**
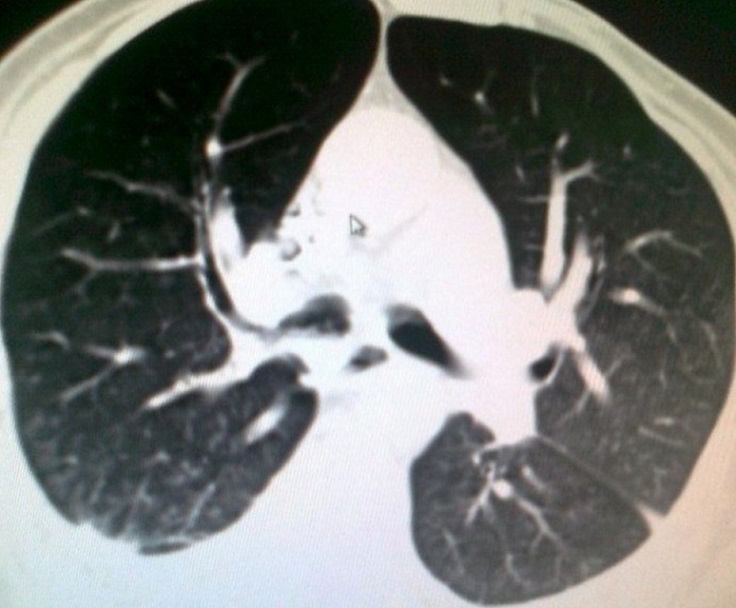
Well developed right upper lobe bronchus with hyper-expansion

**Figure 4:**
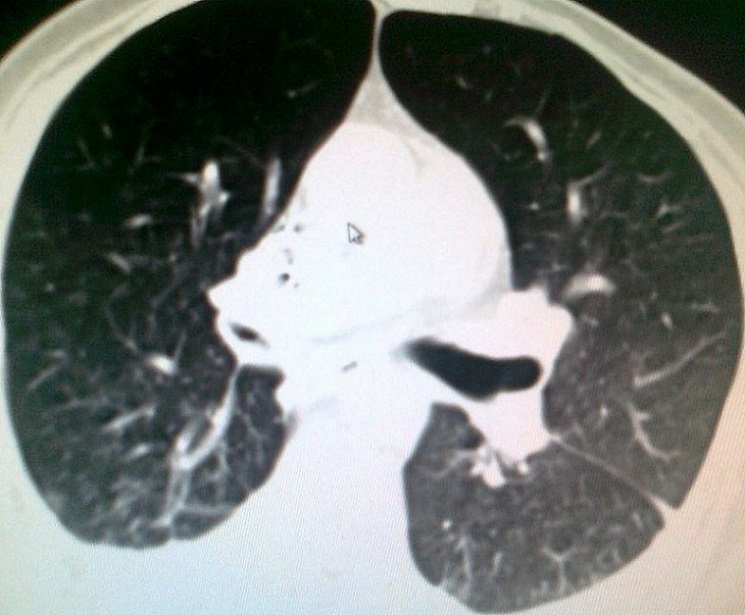
Marked narrowing of the right main bronchus with non-negotiable terminal end

**Figure 5:**
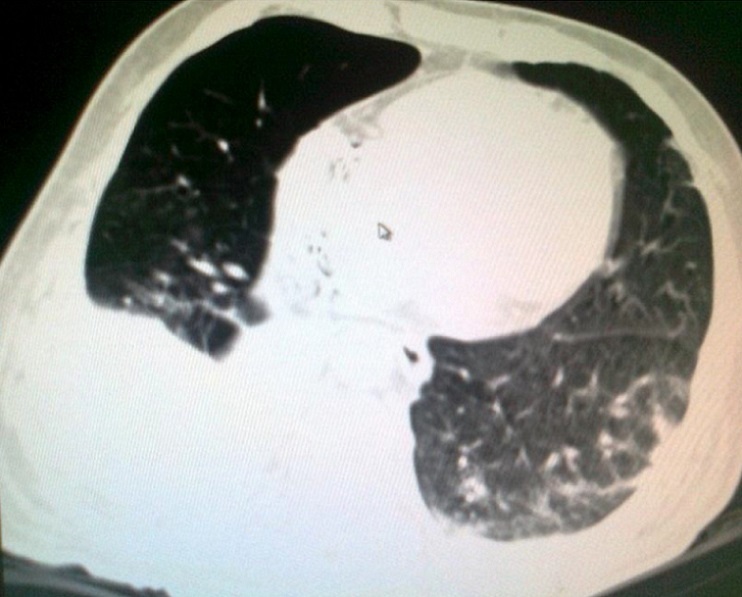
Hyper-expansion of the right upper lobe occupying most of the right upper hemithorax with right lower hemithorax opacity due to bilobar atresia. Signs of inflammation and minimal pl.effusion on left side

**Figure 6:**
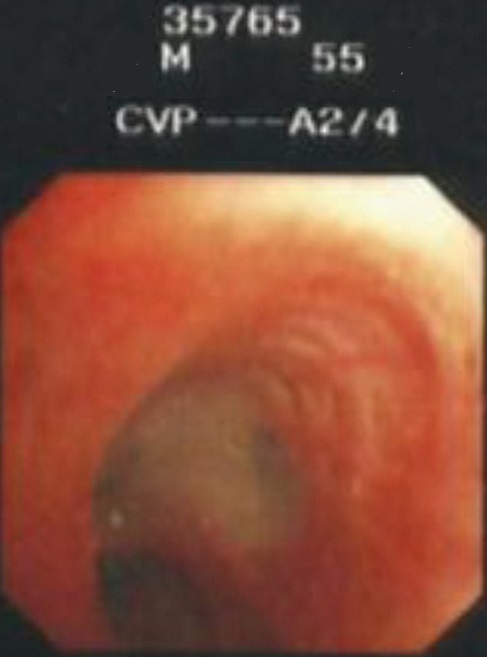
Carina is rotated anti-clockwise due to hyperexpansion of right upper lobe and excessive greenish secretions are visible at carina filling right main bronchus enternace due to pseudomonas aeroginosa

**Figure 7:**
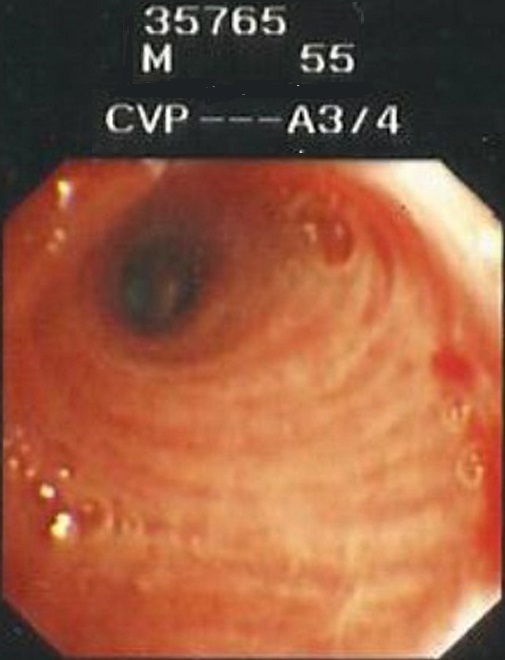
Distant narrowed right main bronchus with blind, non-negotiable terminal end of intermediate bronchus after washing of secretions showing signs of chronic inflamation

A 54 years Yemeni male admitted for rehabilitation after sustaining battle field gun injury complicated with quadriplegia was referred for pulmonary consultation on admission to HDU for having fever, abnormal chest x-ray and respiratory failure arterial blood gases (ABG). PH = 7.36 and PaCo2 = 45.6 and PaO2 = 54.8 BE = -5.9, bicorbanate =26.2. General examination was remarkable for temperature 38.5C0, shortness of breath and episodic cardiac tachy-brady arrythmias. Chest examination revealed central trachea, decreased movements on the right side, impaired percussion note, and ipsilateral absent breath sounds with regional crackles and expiratory wheez. Left side was unremarkable apart from having crackles at the lower chest and expiratory wheez. Blood examination showed leukocytosis and neutrophilia. Bacterial Culture on bronchoscpic alveolar lavage reported heavy growth of Pseudomonas aeroginsoa. Patient was treated under initial diagnosis of right sided pneumonia with a combination of two antipseudomonal antibiotics. His fever subsided, respiratory insufficiency improved as well but the opacity on the right side remained un-resolved. Patient had past history of treatment for bronchial asthma. Chest x-rays (Figure 1) reported opacity in the right hemithorax with volume loss and ipsilateral shifted heart shadow. He was further investigated by contrast CT-Scan chest showing right sided well-defined opacity involving right middle and lower zone, well developed right upper lobe and narrow right main bronchus with absent right middle and lower lobe feeding bronchi and vessels (Figure 2, Figure 3, Figure 4, Figure 5). Bronchoscopy showing anti clock wise rotation of the carina, creamy greenish purulent thick secretions seen at the entrance of right main bronchus and very narrow intermediate bronchus with non-negotiable blind end. (Figure 6, Figure 7). Following episodic tachy-bradycardia, based on electrophysiological studies he was diagnosed having pre-mature sick sinus syndrome as cause of conduction defects requiring a permanent pacemaker (in Figure 1). On further systemic review patent was discovered having right ureteric atresia requiring a stent associated with hydronephrosis of the ipsilateral kidney and a urinary bladder stone. There was past history of breathlessness and wheezing requiring use of inhalers and occasional antibiotics. There was no history of pulmonary tuberculosis and no history of contact. On spirometry vital capacity = 3.29 (69%), FVC = 3.39 (70%), FEV1 = 2.46 (68%), FEV1/FVC = 72%, significant post bronchodilator response of 31.7%. A diagnosis of right lung bi-lobar agenesis with multi system involvement and having mixed restrictive and obstructive airway disease was made.

## Discussion

Pulmonary agenesis is classified in to three types by Schneider [10] in 1912; a-Agenesis-Complete absence of lung and bronchus and no vascular supply to the affected side. b-Aplasia-Rudimentary bronchus with complete absence of pulmonary parenchyma c-Hypoplasia-Presence of variable amount of bronchial tree, pulmonary parenchymal and supportive vasculature. According to Spencer since 1977 [11] pulmonary agenesis is divided as follows; **1-Bilateral complete agenesis**. **2-Unilateral agenesis**: 2 (a)Complete absence of bronchi, 2 (b)Rudimentary bronchus present but no pulmonary parenchyma Or 2 (c)Poorly developed main bronchus with poorly organized parenchyma. **3-Lobar agenesis**. Exact etiology of this condition is not known. Autosomal recessive mode of inheritance is said to play a role. Incidence is greater in males than females in adults, 1.2 cases/100,000 male cases. The age of presentation is variable but usually the condition is diagnosed during 2nd and 3rd decade of life.

In children females dominate (59%), female to male ratio 2:1. These patients are known to have variable symptoms, often there is history of recurrent respiratory infections as in our case. Association of bronchial asthma is a recognized feature. About 50% of patients are reported to have multisystem involvement such as cardiovascular, urogenital, gastrointestinal and skeletal system [11–13]. The subject patient would be classified as type 3-lobar agenesis, besides having cardiovascular as well as urological system abnormalities and mixed restrictive and obstructive airway disease.

Differential diagnosis usually includes pneumonia, collapse, empyema, destroyed lung or thickened pleura. CT-Scan chest with contrast and bronchoscopy are important tools of diagnosis in large majority of patients besides MRI angiography. This condition is usually diagnosed at an early age (mean age 17 years) with history of recurrent or chronic respiratory symptoms particularly if associated with abnormal chest radiographs showing non-resolving opacities. Old age patients although less in number but are no exception as the subject patient. Prognosis depends upon residual normal lung function and the side affected. Left lung agenesis is commoner than right lung, those with left lung agenesis has better survivals than those with right lung involvement or having multisystem abnormalities [14]. Management is mainly conservative and surgery in minority of selected cases.

## Conclusion

Agenesis of lung must be kept in mind while managing patients having non-resolving lung opacities irrespective of their age. Patients typically have history of recurrent chest infections and respiratory symptoms. Multi-system involvement although very rare, but is a recognized feature as in our patient.
